# Associations of the baseline level and change in glycosylated hemoglobin A1c with incident hypertension in non-diabetic individuals: a 3-year cohort study

**DOI:** 10.1186/s13098-022-00827-8

**Published:** 2022-04-18

**Authors:** Lijuan Liu, Donghu Zhen, Songbo Fu, Weiming Sun, Hongli Li, Nan Zhao, Lijie Hou, Xulei Tang

**Affiliations:** Department of Endocrinology, The First Hospital of Lanzhou University, Lanzhou, Gansu China

**Keywords:** Glycosylated hemoglobin A1c, Blood pressure, Hypertension, Cohort

## Abstract

**Background:**

Diabetes mellitus increases the risk of developing hypertension. The relationship between glycosylated hemoglobin A1c (HbA1c) level and incident hypertension remains controversial. This study examined the associations of the baseline level and change in the HbA1c level over 3 years with incident hypertension in non-diabetic individuals.

**Methods:**

This community-based cohort study was conducted with 2591 individuals aged 40–75 years without hypertension or diabetes at baseline, who participated in a longitudinal (REACTION) study program. Questionnaires were administered during interviews, and anthropometric and laboratory measurements were performed at baseline (2011) and follow-up (2014–2015). Multivariate logistic regression models were applied to estimate odds ratios (ORs) and 95% confidence intervals (CIs) of incident hypertension.

**Results:**

Over a median follow-up period of 3.08 years (interquartile range 3.00, 3.25), 384 (14.82%) subjects developed hypertension. In the fully adjusted linear regression models, change in HbA1c remained significantly associated with changes in systolic blood pressure and diastolic blood pressure [β-coefficient (95% CI), 4.421 (2.811–6.032), 1.681 (0.695–2.667)]. Logistic regression analyses showed that baseline HbA1c level was positively associated with incident hypertension in the unadjusted model; however, the association was no longer significant after further adjustment. Change in HbA1c was positively associated with the development of hypertension, both as a categorical variable stratified by tertiles [adjusted OR (95% CI) in the highest tertile was 1.690 (1.240–2.303) versus the lowest tertile)] and as a continuous variable [adjusted OR (95% CI), 1.242 (1.106–1.394)], independent of age, sex, body mass index, systolic blood pressure, fasting plasma glucose level, lipid profile, the HbA1c level at baseline and 3-year change in body mass index.

**Conclusions:**

A higher baseline HbA1c level was not an independent risk factor for incident hypertension, whereas the change in HbA1c was independently associated with a greater longitudinal increase in blood pressure and an increased risk of incident hypertension in non-diabetic individuals.

## Background

Hypertension presents a global public health challenge because of its high prevalence and its associations with cardiovascular disease and all-cause mortality [1]. Hypertension and diabetes tend to cluster in the same individuals and share similar pathophysiological mechanisms, including but not limited to obesity, insulin resistance, hyperinsulinemia, endothelial cell dysfunction, and oxidative stress [2]. A population-based prospective cohort study showed that type 2 diabetes mellitus may causally affect hypertension [3].

Glycated hemoglobin A1c (HbA1c), which reflects the average blood glucose level over the preceding 3 months, is used to monitor glycaemic control and has been added to the diagnostic criteria for diabetes [4, 5]. HbA1c levels at baseline also showed strong associations with incident diabetes and diabetes-related complications in non-diabetic adults [6]. Recently, the role of HbA1c outside of diabetes has received increasing attention. It has been shown that the HbA1c level is significantly associated with the risk of cardiovascular disease even in non-diabetic populations [7, 8]. In addition, HbA1c is a reliable risk factor for all-cause and cardiovascular mortality in both diabetic and non-diabetic populations [8, 9]. Recent studies have attempted to extend the role of HbA1c by including it as a useful component of the definition of metabolic syndrome [10, 11].

Whether HbA1c levels promote hypertension remains controversial. Cross-sectional surveys have shown that elevated HbA1c levels are associated with the prevalence of hypertension, even in non-diabetic populations [12, 13]. To date, there have been several longitudinal studies that investigated the association between baseline HbA1c level and future hypertension risk. A community-based prospective study conducted with 9603 middle-aged participants showed that higher baseline HbA1c values were associated with an increased risk of hypertension independent of known risk factors in subjects with and without diabetes [14]. The Women’s Health Study showed that the HbA1c level in women without diabetes, was associated with an increased risk of hypertension after controlling for most traditional hypertension risk factors, but an additional adjustment for BMI eliminated the relationship [15]. Several studies showed that elevated concentrations of FPG, rather than HbA1c, were significantly predictive of future hypertension at follow-up [16–18]. Notably, previous cohort studies only analysed the association between baseline HbA1c level and incident hypertension and did not consider the impact of dynamic changes in the HbA1c level on hypertension during follow-up. Recent studies showed that in addition to baseline values, changes in classic risk factors, including serum uric acid concentration, body weight, body mass index (BMI), and waist circumference, were significantly associated with an increased risk of incident hypertension [19–22]. Furthermore, little is known about the change in HbA1c over time and its association with the development of hypertension in non-diabetic populations. Therefore, we prospectively investigated the associations of the baseline HbA1c level and the 3-year change in HbA1c level with the risk of incident hypertension among middle-aged and elderly subjects without hypertension or diabetes at baseline.

## Methods

### Study design and participants

The present study was a part of the Risk Evaluation of cAncers in Chinese diabeTic Individuals: a lONgitudinal (REACTION) study [23, 24]. A total of 10,100 residents (2942 men and 7158 women) aged 40–75 years from three communities in Lanzhou City, Northwest China, were randomly identified and underwent examinations at their community hospital from May to September 2011. Among these subjects, we excluded individuals with any of the following: (1) missing blood samples, information on questionnaires or blood pressure values at baseline (n = 229); (2) a history of hypertension or the use of antihypertensive medications (n = 2357), a systolic blood pressure (SBP) or diastolic blood pressure (DBP) higher than 140 mmHg or 90 mmHg, respectively (n = 1381); (3) a history of diabetes or the use of antidiabetic medications (n = 1161), a fasting plasma glucose (FPG) level ≥ 7.0 mmol/L, a 2-h plasma glucose (2hPG) level ≥ 11.1 mmol/L (n = 1326), and an HbA1c level ≥ 6.5% (n = 399). Finally, given that some excluded subjects met more than one exclusion criterion, a group of 4843 subjects without hypertension or diabetes at baseline was identified. A 3-year follow-up survey was carried out from June 2014 to July 2015, which included 2620 subjects who participated on-site, 1238 subjects who completed a telephone survey, 63 subjects who died, and 922 subjects who were lost to follow-up. We limited our analysis to individuals who participated in the follow-up on-site. After the further exclusion of participants with missing HbA1c values or blood pressure data (n = 5) and those who were diagnosed with diabetes or started using antidiabetic medications in the period between the baseline and follow-up surveys (n = 24), 2591 participants were included in the final analyses as the eligible study population.

### Ethics approval and consent to participate

All procedures in this study were approved by the Medical Ethics Committee of Ruijin Hospital, Shanghai Jiaotong University and the Ethics Committee of the First Hospital of Lanzhou University. All subjects provided informed consent prior to their inclusion. As part of the informed consent process, information on the study, the participants' rights as research subjects, and the benefits and possible risks involved were clearly explained. Privacy and confidentiality of the data collected from participants were ensured by using codes to identify participants' completed questionnaires and specimens.

### Data collection

Questionnaires were administered during interviews, and anthropometric and laboratory measurements were performed by trained research staff at baseline and follow-up. General information on education level, smoking status, alcohol consumption, major chronic disease history, and medication history was obtained during interviews using a standardized questionnaire. Body weight was measured to the nearest 0.1 kg using a digital scale with the subject wearing light clothing and no shoes. Height was measured to the nearest 0.1 cm. BMI was calculated as weight in kilograms divided by height in meters squared (kg/m^2^). Blood pressure was measured with standard mercury sphygmomanometers with the subject in a seated position after a period of seated rest. The blood pressure measurements were performed three times, and the average value was used for the analysis. Venous blood samples were taken in the morning following overnight fasting for at least 10 h. All participants underwent a standard 75-g oral glucose tolerance test or standard meal test. FPG and 2hPG concentrations were measured using the glucose oxidase method within 2 h after blood sample collection under stringent quality control. The HbA1c level was measured by high-performance liquid chromatography. Triglycerides, total cholesterol, high-density lipoprotein cholesterol (HDL-C), and low-density lipoprotein cholesterol (LDL-C) were measured by chemiluminescence on an autoanalyzer.

### Definitions of variables and outcomes

On the baseline and follow-up surveys, hypertension was defined as an SBP ≥ 140 mmHg and/or a DBP ≥ 90 mmHg and/or a self-reported history of hypertension and the current use of antihypertensive medications [25]. Outcomes at the 3-year follow-up were incident hypertension and changes in SBP and DBP.

Diabetes was defined as meeting one of the following criteria: (1) an FPG level ≥ 7.0 mmol/L; (2) a 2hPG level ≥ 11.1 mmol/L; (3) an HbA1c level ≥ 6.5%; (4) a previous diagnosis of diabetes; (5) the use of drug treatment for diabetes or the regular injection of insulin [5].

A current smoker was defined as a person who smoked cigarettes daily or occasionally. A current alcohol consumer was defined as a person who drank alcohol regularly in the past 6 months. Higher education was defined as having attended junior college or higher.

The changes in various factors were calculated as the value at follow-up minus the value at baseline. Baseline serum HbA1c levels (%) were divided into tertiles (4.0–5.6%, 5.7–5.9%, 6.0–6.4%). Change in HbA1c level was divided into tertiles (≤ − 0.4%, − 0.3 to − 0.2%, ≥ − 0.1%).

### Statistical analysis

Continuous variables are described as the mean ± standard deviation (SD), and categorical variables are described as numbers (percent). Baseline and changes in parameters over 3 years were compared according to the presence or absence of hypertension by using Student’s t-test or the Mann–Whitney test for continuous variables and the chi-square test for categorical variables. Trends of variables among the groups categorized by the HbA1c level at baseline and change in the HbA1c level, stratified by tertiles, were analyzed by linear regression for the continuous variables (as the median value in each tertile) and linear-by-linear analysis using the chi-square test for categorical variables, respectively.

The associations of baseline HbA1c level and change in HbA1c level with changes in SBP and DBP over the 3-year period were assessed by linear regression, with changes in SBP and DBP as the dependent variables and baseline HbA1c and change in HbA1c as the independent variables; β-coefficients and 95% confidence intervals (CIs) were calculated. Logistic regression analysis of the risk of developing hypertension was performed using the baseline HbA1c level or change in the HbA1c level stratified by tertiles or as continuous variables (1-SD increment) as the independent variables. The association of the combined effect of baseline HbA1c and change in the HbA1c level with incident hypertension was evaluated by multiple logistic regression analysis, with incident hypertension as the dependent variable and the combined tertiles of baseline HbA1c and change in HbA1c as the independent variables; odds ratios (ORs) and 95% CIs were calculated. Covariates that were considered as potential confounders were included in the following multivariable regression models: Model 1: Adjusted for age (continuous), sex, education level, current smoking, and current alcohol consumption; Model 2: Further adjusted for BMI, FPG, total cholesterol, triglycerides, HDL-C, and SBP at baseline (all continuous); Model 3: Further adjusted for change in BMI (continuous); Model 4: Further adjusted for the HbA1c level at baseline (continuous). A *p*-value < 0.05 was considered significant. All analyses were performed using SPSS version 22.0 for Windows (SPSS Inc., Chicago, IL, USA).

## Results

### General characteristics

A total of 2591 participants (males, n = 568) aged 40–75 years without hypertension or diabetes at baseline were included in the final analysis. The mean age at baseline was 55.26 ± 7.35 years. At 3.08 years (interquartile range 3.00, 3.25) after the baseline examination, 384 participants had developed hypertension (incidence rate 14.82%).

When general characteristics were compared according to hypertension status at follow-up, the individuals who had developed hypertension were older and had a significantly higher BMI, SBP, DBP, HbA1c level, FPG level, 2hPG level, triglyceride level, and HDL-C level at baseline; in addition, those who developed hypertension also had significantly greater changes in HbA1c level, SBP and DBP over 3 years than those who did not develop hypertension (*p* < 0.05) (Table 1).

**Table 1:** Characteristics of the total study participants and according to the incidence of hypertension at 3-year follow-up

Characteristics	Total	Incident hypertension		*p* value
	(N = 2591)	No (N = 2207)	Yes (N = 384)	
*Baseline parameters*				
Age (years)	55.26 ± 7.35	54.82 ± 7.26	57.77 ± 7.37	< 0.001
Men, n (%)	568 (21.9)	471 (21.3)	97 (25.3)	0.095
BMI (kg/m^2^)	23.44 ± 4.02	23.29 ± 4.13	24.32 ± 3.18	< 0.001
SBP (mmHg)	116.8 ± 14.8	115.3 ± 13.9	125.4 ± 17.0	< 0.001
DBP (mmHg)	71.9 ± 9.9	71.2 ± 9.5	75.8 ± 11.6	< 0.001
HbA1c (%)	5.77 ± 0.35	5.76 ± 0.35	5.82 ± 0.34	0.003
FPG (mmol/L)	5.32 ± 0.60	5.29 ± 0.59	5.44 ± 0.63	< 0.001
2hPG (mmol/L)	6.75 ± 1.65	6.67 ± 1.62	7.24 ± 1.73	< 0.001
Total cholesterol (mmol/L)	4.56 ± 1.00	4.55 ± 0.99	4.63 ± 1.04	0.09
Triglycerides (mmol/L)	1.61 ± 0.99	1.59 ± 0.97	1.75 ± 1.10	0.005
LDL- C (mmol/L)	2.56 ± 0.75	2.55 ± 0.75	2.62 ± 0.77	0.098
HDL-C (mmol/L)	1.27 ± 0.31	1.28 ± 0.30	1.24 ± 0.31	0.048
Higher education, n (%)	428 (16.5)	366 (16.6)	62 (16.1)	0.882
Current smoker, n (%)	278 (10.7)	235 (10.6)	43 (11.2)	0.722
Current alcohol consumer, n (%)	110 (4.2)	91 (4.1)	19 (4.9)	0.492
*Changes in parameters over 3 years*				
Change in HbA1c	− 0.24 ± 0.31	− 0.25 ± 0.30	− 0.19 ± 0.35	0.001
Change in BMI	0.43 ± 3.45	0.37 ± 3.67	0.73 ± 1.74	0.065
Change in SBP	4.27 ± 15.81	2.30 ± 13.89	15.61 ± 20.68	< 0.001
Change in DBP	1.64 ± 9.87	0.83 ± 9.16	6.30 ± 12.25	< 0.001

Data are presented as means ± standard deviation, or numbers (percent). *BMI* body mass index, *SBP* systolic blood pressure, *DBP* diastolic blood pressure, *HbA1c* glycosylated hemoglobin A1c, *FPG* fasting plasma glucose, *2hPG* 2-h plasma glucose, *LDL-C* low-density lipoprotein cholesterol, *HDL-C* high-density lipoprotein cholesterol

### Characteristics of the subjects categorized according to the tertiles of baseline HbA1c and change in HbA1c

The mean HbA1c values ± SD at baseline and follow-up were 5.77 ± 0.35% and 5.53 ± 0.37%, respectively. The mean change in the HbA1c level was -0.24 ± 0.31%. At the 3-year follow-up, 84 subjects were newly diagnosed with diabetes, 27 (32.1%) of whom had developed hypertension. The characteristics of the subjects categorized according to the tertiles of baseline HbA1c or change in HbA1c are shown in Table 2. In general, the incidence rate of hypertension appeared to increase as the tertile of baseline HbA1c and change in HbA1c increased (*p for trend* < 0.01). Age, BMI, the FPG level, the 2hPG level, the total cholesterol level, and the LDL-C level at baseline were significantly higher and the change in HbA1c was significantly smaller in those with values in the higher baseline HbA1c level tertiles (*p for trend* < 0.05). The baseline triglyceride level and changes in SBP, DBP, and BMI were significantly higher, but age, the FPG level, and the HbA1c level at baseline were significantly lower in those with values in the higher tertiles of change in HbA1c (*p for trend* < 0.01) (Table 2).

**Table 2:** Characteristics of the subjects categorized according to tertiles of baseline HbA1c level and change in HbA1c

Characteristics, N = 2591	Tertiles of baseline HbA1c (%)				Tertiles of change in HbA1c (%)			
	Tertile1 (4.0–5.6) (N = 892)	Tertile2 (5.7–5.9) (N = 904)	Tertile3 (6.0–6.4) (N = 795)	*p* for trend	Tertile1 (≤ − 0.4) (N = 966)	Tertile2 (− 0.3 to − 0.2) (N = 750)	Tertile3 (≥ − 0.1) (N = 875)	*p* for trend
Incident hypertension, n (%)	107 (12.0)	138 (15.3)	139 (17.5)	0.001	119 (12.3)	117 (15.6)	148 (16.9)	0.005
Baseline parameters								
Age (years)	52.77 ± 7.45	55.67 ± 7.13	57.52 ± 6.77	< 0.001	55.97 ± 7.12	55.19 ± 7.45	54.54 ± 7.45	< 0.001
Men, n (%)	196 (22.0)	200 (22.1)	172 (21.6)	0.872	217 (22.5)	163 (21.7)	188 (21.5)	0.610
BMI (Kg/m^2^)	23.31 ± 5.19	23.30 ± 3.08	23.76 ± 3.39	0.028	23.24 ± 2.85	23.54 ± 5.77	23.59 ± 3.23	0.061
SBP (mmHg)	116.5 ± 13.1	116.0 ± 16.6	118.0 ± 14.4	0.051	117.2 ± 14.1	116.3 ± 15.7	116.7 ± 14.8	0.499
DBP (mmHg)	72.2 ± 9.2	71.5 ± 10.9	71.9 ± 9.7	0.485	72.1 ± 9.6	71.7 ± 10.5	71.9 ± 9.9	0.650
HbA1c (%)	5.39 ± 0.25	5.80 ± 0.08	6.15 ± 0.13	< 0.001	5.91 ± 0.29	5.76 ± 0.30	5.61 ± 0.38	< 0.001
FPG (mmol/L)	5.14 ± 0.59	5.32 ± 0.59	5.51 ± 0.58	< 0.001	5.37 ± 0.59	5.31 ± 0.62	5.26 ± 0.61	< 0.001
2hPG (mmol/L)	6.35 ± 1.54	6.73 ± 1.61	7.23 ± 1.69	< 0.001	6.81 ± 1.62	6.68 ± 1.64	6.76 ± 1.68	0.475
Total cholesterol (mmol/L)	4.38 ± 0.93	4.55 ± 0.97	4.77 ± 1.06	< 0.001	4.52 ± 1.02	4.54 ± 1.00	4.61 ± 0.97	0.058
Triglycerides, (mmol/L)	1.58 ± 1.02	1.60 ± 0.93	1.66 ± 1.02	0.105	1.54 ± 0.93	1.59 ± 0.95	1.71 ± 1.08	0.001
LDL-C (mmol/L)	2.41 ± 0.69	2.55 ± 0.74	2.74 ± 0.79	< 0.001	2.54 ± 0.75	2.55 ± 0.75	2.60 ± 0.76	0.139
HDL-C (mmol/L)	1.27 ± 0.30	1.27 ± 0.31	1.27 ± 0.31	0.928	1.27 ± 0.32	1.27 ± 0.30	1.27 ± 0.29	0.988
Higher education, n (%)	157 (17.6)	142 (15.7)	129 (16.2)	0.432	156 (16.1)	114 (15.2)	158 (18.1)	0.285
Current smoker, n (%)	99 (11.1)	89 (9.8)	90 (11.3)	0.912	108 (11.2)	73 (9.7)	97 (11.1)	0.926
Current alcohol consumer, n (%)	47 (5.3)	28 (3.1)	35 (4.4)	0.341	45 (4.7)	28 (3.7)	37 (4.2)	0.632
Changes in parameters over 3 years								
Change in HbA1c	− 0.11 ± 0.32	− 0.27 ± 0.25	− 0.35 ± 0.30	< 0.001	− 0.52 ± 0.15	− 0.25 ± 0.05	0.08 ± 0.26	< 0.001
Change in BMI	0.43 ± 5.01	0.50 ± 2.08	0.33 ± 2.39	0.561	0.31 ± 1.87	0.19 ± 5.25	0.76 ± 2.77	0.007
Change in SBP	3.89 ± 13.22	5.10 ± 17.40	3.75 ± 16.54	0.902	2.65 ± 14.73	4.44 ± 17.60	5.91 ± 15.18	< 0.001
Change in DBP	1.45 ± 8.73	2.01 ± 10.92	1.43 ± 9.81	0.994	0.89 ± 9.23	1.77 ± 10.85	2.35 ± 9.63	0.002

Data are presented as means ± standard deviation, or numbers (percent). *p* value for trend from linear regression for continuous variables or linear-by-linear analysis from chi-square tests for categorical variables. *BMI* body mass index, *SBP* systolic blood pressure, *DBP* diastolic blood pressure, *HbA1c* glycosylated hemoglobin A1c, *FPG* fasting plasma glucose, *2hPG* 2-h plasma glucose, *LDL-C* low-density lipoprotein cholesterol, *HDL-C* high-density lipoprotein cholesterol

### Associations of baseline HbA1c and change in HbA1c with changes in SBP and DBP

The average changes in SBP and DBP during follow-up were 4.27 ± 15.81 and 1.64 ± 9.87 mmHg, respectively. In the linear regression models with the changes in SBP or DBP as the dependent variables, an unadjusted positive relationship was identified between baseline HbA1c and change in SBP; however, the association between baseline HbA1c and change in SBP was not significant after further adjustment in Models 1–3. Change in HbA1c was significantly and positively associated with changes in SBP and DBP in the crude analyses. After adjustment for sex, age, current smoking, current alcohol consumption, education level, baseline levels of total cholesterol, triglycerides, HDL-C, and blood pressure, baseline BMI, change in BMI, and baseline HbA1c in Models 1–4, change in HbA1c remained significantly associated with changes in SBP and DBP (*p* < 0.001). Each one-unit (%) increment in the change in HbA1c over 3 years was associated with a 4.421-mmHg change in SBP and a 1.681-mmHg change in DBP (Table 3).

**Table 3:** Association of baseline and change in HbA1c with changes in SBP and DBP

	Change in SBP		Change in DBP*	
	β-coefficient (95% CI)	p value	β-coefficient (95% CI)	p value
*Baseline HbA1c*				
Unadjusted	1.971 (0.105–3.837)	0.038	0.984 (− 0.184–2.152)	0.099
Model 1	− 0.538 (− 2.340–1.265)	0.599	0.246 (− 0.877–1.369)	0.668
Model 2	− 0.042 (− 0.617–1.032)	0.881	− 0.168 (− 1.035–0.699)	0.704
Model 3	− 0.023 (− 1.501–1.455)	0.976	− 0.243 (− 1.103–0.617)	0.580
*Change in HbA1c*				
Unadjusted	5.528 (3.415–7.642)	< 0.001	2.328 (1.005–3.651)	0.001
Model 1	4.996 (3.033–6.958)	< 0.001	1.978 (0.753–3.204)	0.002
Model 2	5.020 (3.528–6.512)	< 0.001	1.830 (0.919–2.742)	< 0.001
Model 3	4.473 (2.989–5.957)	< 0.001	1.527 (0.619–2.436)	0.001
Model 4	4.421 (2.811–6.032)	< 0.001	1.681 (0.695–2.667)	0.001

Data are *β*-coefficients of linear regression (95% confidence interval). Model 1: adjusted for age (continuous), sex, education level, current smoking, and current alcohol consumption; Model 2: Model 1 + body mass index, fasting plasma glucose, total cholesterol, triglycerides, high-density lipoprotein cholesterol, and SBP at baseline (all continuous); Model 3: Model 2 + change in body mass index (continuous); Model 4: Model 3 + HbA1c at baseline (continuous). * In Models 2–4 with change in DBP as the dependent variable, DBP at baseline instead of SBP at baseline was adjusted. *CI* confidence interval; *HbA1c* glycated hemoglobin A1c; *SBP* systolic blood pressure; *DBP* diastolic blood pressure

### Association of baseline HbA1c and change in HbA1c with the risk of incident hypertension

Logistic regression analyses were used to assess the associations of baseline level of HbA1c and change in HbA1c with the risk of incident hypertension. The baseline HbA1c level, both stratified into tertiles and as a continuous variable (per 1-SD increment), was positively associated with incident hypertension in the unadjusted model; however, the association lost significance after adjustments in Model 1, which was adjusted for age, sex, education level, current smoking and current alcohol consumption; Model 2, which was further adjusted for the baseline levels of BMI, SBP, FPG, total cholesterol, triglycerides, and HDL-C; and Model 3, which was further adjusted for change in BMI over 3 years.

Changes in the HbA1c level as tertiles and continuous values (per 1-SD increment) were positively associated with the development of hypertension, independent of age, sex, education level, current smoking, current alcohol consumption, BMI, SBP, FPG, total cholesterol, triglycerides, and HDL-C levels at baseline, and change in BMI over 3 years, even baseline HbA1c (Models 1–4) (Table 4).

**Table 4:** Association of baseline and change in HbA1c with risk of incident hypertension

	Odds ratio (95% CI) of tertiles				Odds ratio (95% CI) of continuous value	
	Tertile1	Tertile2	Tertile3	*p* value	per 1-SD increment	*p* value
*Baseline HbA1c*						
Unadjusted	1 (ref)	1.322 (1.008–1.734)	1.555 (1.184–2.042)	0.011	1.186 (1.061–1.324)	0.003
Model 1	1 (ref)	1.165 (0.883–1.536)	1.250 (0.943–1.658)	0.291	1.090 (0.973–1.222)	0.138
Model 2	1 (ref)	1.157 (0.856–1.565)	1.109 (0.808–1.523)	0.632	1.011 (0.891–1.147)	0.866
Model 3	1 (ref)	1.155 (0.853–1.565)	1.073 (0.779–1.479)	0.644	0.998 (0.878–1.133)	0.971
*Change in HbA1c*						
Unadjusted	1 (ref)	1.316 (0.999–1.732)	1.449 (1.116–1.881)	0.017	1.187 (1.075–1.310)	0.001
Model 1	1 (ref)	1.375 (1.041–1.817)	1.576 (1.209–2.053)	0.003	1.231 (1.114–1.361)	< 0.001
Model 2	1 (ref)	1.505 (1.114–2.034)	1.686 (1.263–2.251)	0.001	1.242 (1.115–1.384)	< 0.001
Model 3	1 (ref)	1.512 (1.117–2.047)	1.598 (1.193–2.140)	0.003	1.214 (1.088–1.354)	< 0.001
Model 4	1 (ref)	1.551 (1.142–2.107)	1.690 (1.240–2.303)	0.002	1.242 (1.106–1.394)	< 0.001

Model 1: adjusted for age (continuous), sex, education level, current smoking, current alcohol consumption; Model 2: Model 1 + body mass index, fasting plasma glucose, total cholesterol, triglycerides, high-density lipoprotein cholesterol, and systolic blood pressure at baseline (all continuous); Model 3: Model 2 + change in body mass index (continuous); Model 4: Model 3 + HbA1c at baseline (continuous). *CI* confidence interval, *HbA1c* glycated hemoglobin A1c

### Association of the combination of the tertiles of baseline HbA1c and change in HbA1c with incident hypertension

To prospectively investigate whether the combination of the tertiles of baseline HbA1c level and change in HbA1c was associated with incident hypertension, logistic regression analyses were performed. The unadjusted and adjusted ORs (in Models 1–3) showed that higher tertiles of the combination of the baseline HbA1c level and change in HbA1c were significant and independent determinants of incident hypertension (*p* < 0.01) (Table 5).

**Table 5:** Combining tertiles of baseline and change in HbA1c for incident hypertension

Baseline HbA1c (%)	Change in HbA1c	N	n (%)	Unadjusted OR (95% CI)	Adjusted OR (95% CI)		
					Model 1	Model 2	Model 3
Tertile1 (4.0–5.6) (N = 892)	Tertile1 (≤ − 0.4)	168	15 (8.9)	1 (ref)	1 (ref)	1 (ref)	1 (ref)
	Tertile2 (− 0.3–0.2)	260	33 (12.7)	1.483 (0.779–2.823)	1.550 (0.810–2.967)	1.721 (0.867–3.415)	1.757 (0.883–3.497)
	Tertile3 (≥ − 0.1)	464	59 (12.7)	1.486 (0.818–2.698)	1.484 (0.813–2.708)	1.505 (0.797–2.843)	1.444 (0.762–2.738)
Tertile2 (5.7–5.9) (N = 904)	Tertile1 (≤ − 0.4)	357	45 (12.6)	1.471 (0.795–2.723)	1.317 (0.708–2.452)	1.216 (0.629–2.351)	1.221 (0.630–2.365)
	Tertile2 (− 0.3–0.2)	289	48 (16.6)	2.032 (0.897–1.630)	1.777 (0.955–3.308)	1.953 (1.008–3.784)	1.944 (1.001–3.775)
	Tertile3 (≥ − 0.1)	258	45 (17.4)	2.155 (1.159–4.007)	1.950 (1.042–3.649)	2.196 (1.126–4.284)	2.057 (1.051–4.028)
Tertile3 (6.0–6.4) (N = 795)	Tertile1 (≤ − 0.4)	441	59 (13.4)	1.575 (0.867–2.862)	1.274 (0.695–2.334)	1.226 (0.645–2.333)	1.190 (0.623–2.272)
	Tertile2 (− 0.3–0.2)	201	36 (17.9)	2.225 (1.172–4.226)	1.784 (0.931–3.417)	1.626 (0.812–3.256)	1.564 (0.778–3.144)
	Tertile3 (≥ − 0.1)	153	44 (28.8)	4.117 (2.181–7.773)	3.442 (1.808–6.551)	3.063 (1.519–6.175)	2.729 (1.346–5.533)
*p* value				< 0.001	0.001	0.004	0.015

Model 1: adjusted for age (continuous), sex, education level, current smoking, current alcohol consumption; Model 2: Model 1 + body mass index, fasting plasma glucose, total cholesterol, triglycerides, high-density lipoprotein cholesterol, and systolic blood pressure at baseline (all continuous); Model 3: Model 2 + change in body mass index (continuous); *CI* confidence interval, *HbA1c* glycated hemoglobin A1c

## Discussion

In this community-based cohort study conducted among Chinese adults aged 40–75 years old without hypertension or diabetes at baseline, over a median follow-up period of 3.08 years, the incidence of hypertension increased across increasing tertiles of the baseline HbA1c level and change in HbA1c. The association between baseline HbA1c and hypertension was no longer significant after adjusting for possible confounding factors, whereas the change in HbA1c over time was associated with the development of hypertension and a greater longitudinal increase in blood pressure, independent of baseline HbA1c level, change in BMI and well-known hypertension risk factors such as age, sex, BMI, the FPG level, and the lipid profile. Combining the tertiles of the baseline level of HbA1c and change in HbA1c showed that they were significant and independent determinants of incident hypertension.

Several longitudinal reports have demonstrated the relationship between the baseline HbA1c level and incident hypertension, but the conclusions are inconsistent [14–18]. In an urban Northern Chinese population, higher FPG and 2hPG levels, but not HbA1c levels, have been shown to be independently associated with the development of hypertension [18]. In our study, in the crude analyses, baseline HbA1c was a strong predictor of hypertension risk at the 3-year follow-up. However, the predictive value of HbA1c may be attributable to its association with other risk factors, such that the correlation between baseline HbA1c and the development of hypertension lost significance after further adjustment. The inconsistent results of studies on the relationship between the baseline HbA1c level and the risk of developing hypertension may be attributed to the differences in the duration of follow-up and the diversity of HbA1c according to age, sex, and population.

Little evidence exists regarding the association between the change in the HbA1c level over time and the risk of incident hypertension. A prospective study conducted with 5413 Korean participants aged 40–69 years old with a 10-year follow-up period showed that the progression of the glycaemic state was significantly associated with the risk of hypertension; in detail, compared with the maintenance of the glycaemic state, the risk of hypertension proportionally increased in the order of the progression of the glycaemic state from prediabetes to diabetes, from normoglycaemia to prediabetes, and from normoglycaemia to diabetes [26]. The observation in our study suggested that change in HbA1c over 3 years was a better predictor of a higher risk of hypertension and a greater longitudinal increase in blood pressure than the baseline HbA1c value and other major risk factors. A previous report showed that HbA1c levels were associated with age and sex in Chinese adults without a prior diagnosis of diabetes [27]. We considered the possible effects of sex and increasing age on the association between the 3-year change in HbA1c level and hypertension risk by adjusting the model for age and other risk factors. Importantly, although well-known associations have been reported between changes in weight or BMI and changes in blood pressure and the risk for incident hypertension [20, 21], the significant association of the change in HbA1c level with incident hypertension remained, independent of change in BMI in this study. The mechanism remains unclear, but our results suggest that the change in HbA1c might play a direct role in the increase in blood pressure through other mechanisms that are not completely captured by weight gain. The 3-year change in HbA1c was based on the values at baseline and follow-up, so whether the observed change in HbA1c level occurred before or after the development of hypertension remains uncertain. Therefore, the causal relationship between the dynamic change in HbA1c and the risk of hypertension could not be established.

Moreover, our data support the notion that the combination of increased baseline HbA1c level and change in HbA1c was also a significant and independent determinant of incident hypertension, further supporting the relevance of HbA1c for the development of hypertension. Accordingly, 27 cases of incident hypertension occurred in 84 subjects who were newly diagnosed with diabetes in our survey; the incidence rate of hypertension reached 32.1%, higher than that of 14.8% in the total sample. However, individuals with larger increases in their HbA1c levels over 3 years tended to be those with lower baseline HbA1c levels, who were actually at lower risk of hypertension. This might have led to an underestimation of the association between change in HbA1c and the risk of hypertension.

Although the pathogenic mechanisms supporting a plausible link between change in HbA1c and future hypertension risk are not completely understood, several theories have been postulated. First, HbA1c is an indicator predicting insulin resistance. An increase in HbA1c reflects the aggravation of insulin resistance. Homeostasis model assessment of insulin resistance values were associated with HbA1c in subjects without diabetes or obesity [28]. Numerous studies have suggested that insulin resistance is independently associated with an increased risk of hypertension [29, 30]. Insulin resistance may play a role in the pathogenesis of hypertension, potentially via adverse effects on the renin-angiotensin system, sympathetic nervous system, and renal sodium retention [31, 32]. Second, an increased HbA1c level was found to be correlated with biomarkers of inflammation, endothelial dysfunction, and arterial stiffness in a non-diabetic population [33–35]. The KORA S4/F4 study showed that biomarkers of subclinical inflammation, such as high-sensitivity C-reactive protein and serum amyloid A, were independently associated with 7-year changes in HbA1c before the diagnosis of type 2 diabetes [33]. In a general Chinese population, the HbA1c level was positively related to elevated arterial stiffness indices after adjustment for conventional factors, regardless of glucose tolerance status [36]. Third, the HbA1c level is correlated with other metabolic factors that are also proposed to be associated with hypertension. HbA1c values are largely dependent on circulating glucose levels; however, it is possible that non-glycemic factors may be of disproportionate importance in the low range of HbA1c levels. Accordingly, the relationship between HbA1c levels and hypertension might be mediated by underlying diseases or other unknown factors.

The findings of this study also have implications for clinical practice. Change in HbA1c may be a more powerful determinant of hypertension than the baseline HbA1c level, which highlights the importance of monitoring the trend of change in HbA1c levels as a marker of the risk of hypertension in non-diabetic individuals. It has been postulated that long-term glucometabolic status could be an important target for the strategic prevention of hypertension.

To our knowledge, this is the first population-based cohort study to report that change in HbA1c over approximately 3 years was significantly associated with an increased risk of incident hypertension and a greater longitudinal increase in blood pressure in non-diabetic individuals. This association remained significant even after adjustment for FPG level, HbA1c level, lipid profile, BMI, SBP at baseline, and change in BMI. This was also the first study to examine the combined baseline HbA1c level and the change in HbA1c with regard to the prediction of incident hypertension. However, this study has several potential limitations that should be considered when interpreting the results. First, the diagnoses of hypertension at baseline and follow-up were based on measurements taken at a single visit, which could have been influenced by various external factors, leading to unreliable results and the misclassification of blood pressure status. Second, we did not evaluate other factors affecting HbA1c, such as the hemoglobin level, red blood cell abnormalities, and iron storage status [37]. Although we examined the possible confounding effects of other variables on the association between the HbA1c level and the development of hypertension, there remained a possibility that unmeasured factors could have been confounders. Third, a possible limitation is selection bias due to the exclusion of individuals from the follow-up analysis because of incomplete data or loss to follow-up.

In conclusion, an increase in the HbA1c level was significantly associated with a higher risk of incident hypertension and a greater longitudinal increase in blood pressure among apparently healthy adults without hypertension or diabetes at baseline, independent of a large set of covariates, the baseline HbA1c level, and change in BMI, whereas the baseline HbA1c level was not independently associated with the future risk of hypertension. That is, the association between HbA1c and the future hypertension risk could be better explained by the change in HbA1c over time rather than by a single measurement. Attention should be given to the homeostasis of HbA1c levels in non-diabetic adults. However, the causal relationship of the change in HbA1c and hypertension risk was not confirmed. Further prospective and interventional studies are needed to confirm the significant role of change in HbA1c in the development of hypertension and investigate the mechanisms underlying this association.

## Data Availability

The datasets used and analyzed during the current study are available from the corresponding author on reasonable request.

## Abbreviations

BMI: Body mass index
SBP: Systolic blood pressure
DBP: Diastolic blood pressure
HbA1c: Glycosylated hemoglobin A1c
FPG: Fasting plasma glucose
2hPG: 2-Hour plasma glucose
LDL-C: Low-density lipoprotein cholesterol
HDL-C: High-density lipoprotein cholesterol
